# Evaluation of a pressure‐sensitive treadmill in normal cats

**DOI:** 10.1111/jsap.70131

**Published:** 2026-04-03

**Authors:** D. M. Marturello, K. B. de Sousa, B. L. Boger, S. A. Shull, K. L. Perry

**Affiliations:** ^1^ Department of Small Animal Clinical Sciences Michigan State University East Lansing Michigan USA

## Abstract

**Objectives:**

To evaluate a pressure‐sensitive treadmill in normal cats to determine feasibility as a tool for kinetic gait assessment.

**Materials and Methods:**

Institutional ethical approval was obtained. Cats with no evidence of orthopaedic disease were prospectively enrolled and acclimatised to the pressure‐sensitive treadmill. Cats were gaited until three valid trials of 10 gait cycles each were obtained. After normalising to % body weight, peak vertical force, vertical impulse, symmetry index, step phase duration, step length and time for data collection were reported.

**Results:**

Fifty cats with body weights of 4.9 ± 1.1 kg met inclusion criteria. Forelimb peak vertical force and vertical impulse were 57.5 ± 4.2 N and 27.2 ± 3.4 Ns, respectively. Hindlimb peak vertical force and vertical impulse were 46.4 ± 3.8 N and 21 ± 3.1 Ns, respectively. Forelimb and hindlimb symmetry index were 0.99 ± 0.04% and 1.01 ± 0.07%, respectively. Step phase duration was 65.2 ± 3.6% in the forelimbs and 61.2 ± 2.7% in the hindlimbs, while step length was 21.9 ± 2.4 cm and 22.0 ± 2.3 cm in the forelimbs and hindlimbs, respectively. Data collection time was 3.5 ± 1.5 minutes.

**Clinical Significance:**

The pressure‐sensitive treadmill produced consistent and repeatable kinetic data, was easy and fast to use, and cats readily participated in most cases. The homogeneity of protocols using pressure‐sensitive treadmill could allow for useful gait comparisons between studies, facilities and patients in future investigations. This is particularly important in feline literature where case numbers at individual facilities are typically small.

## INTRODUCTION

Gait assessment is one of the primary tools used by veterinarians to localise and diagnose orthopaedic disease. Various methods of gait analysis in small animals have been described to distinguish between normal and abnormal gaits; however, many of these assessments are subjective in nature and are infrequently repeatable, making comparative analysis challenging. Feline patients represent a particularly difficult population for gait evaluation because they do not typically move freely in an unfamiliar environment (Perry, 2014).

Ground reaction forces (GRFs) are well established in describing locomotion and identifying limb asymmetries in small animals. The benefits of objective analyses include reduced observer bias, repeatability and the fact that data can be collected and stored, allowing for comparisons or assessment of patient progression following a given treatment. Two main equipment systems are used for objective gait analysis: force plates (FP) and pressure‐sensitive equipment (walkways or treadmills).

Force plates are transducers that measure force, typically using either strain gauges or piezoelectric sensor transducers (Caldwell et al., 2013). Most investigators prefer multiple in‐line force plates because they permit collection of multiple footfalls as well as reducing both collection time and trial repetition, which is of value in patients with orthopaedic disease (Stejskal et al., 2015). The main benefit of the FP system is the ability to directly record data in three directions: vertical (*z*‐vector), craniocaudal (*y*‐vector) and mediolateral (*x*‐vector), although most investigators have only reported the vertical forces due to their increased magnitude and consistent results (DeCamp, 1997). Disadvantages of FP include (1) lack of portability, (2) large space required for appropriate set‐up and (3) difficulty gaiting small animals with a shorter stride because they frequently have multiple simultaneous paw‐strikes on each plate, which skews the data (Torres, 2018).

Recently, a pressure‐sensitive treadmill (PST) was developed and validated against a force plating system, for use in canine patients (Häusler et al., 2020; Söhnel et al., 2022). In the Söhnel study, investigators found excellent agreement in both peak vertical force (PVF) and vertical impulse (VI), when comparing the PST to traditional FP systems. Furthermore, this group noted an additional benefit to the PST in the form of superior identification of limb asymmetry, because dogs did not have to complete multiple trials with interrupted locomotion while walking back to await the next pass (Söhnel et al., 2022). Lastly, the use of a treadmill system allows for control of both acceleration and velocity outside of a trained handler, parameters which have been shown to significantly affect GRF (Budsberg et al., 1993). Clinically, the data produced by the PST could allow objective assessment of individual patients across time periods and may allow comparisons between studies, hospitals and patients receiving different treatments. This is particularly true for feline patients, where investigators have reported a wide range of speeds used to collect kinetic data with PSW systems (Fowler et al., 1993; Guillot et al., 2013; Lascelles et al., 2007; Romans et al., 2005; Verdugo et al., 2013). Yet, to date, no investigator has evaluated the PST's usefulness in feline patients, a notoriously difficult species to gait information from.

Therefore, the purpose of this descriptive study was to evaluate the commercially available PST system in cats to determine its feasibility as a kinetic assessment tool. Outcome measures included PVF (N), VI (Ns), symmetry index (SI, %), temporospatial parameters (stance phase duration and step length) and time to record three valid trials. Description of our experience using the PST in cats was a secondary aim.

## MATERIALS AND METHODS

### Study design

Institutional approval was obtained (IACUC PROTO202400193). Sixty‐six healthy, owner‐reported normal cats were prospectively enrolled from a population within Michigan State University veterinary community (doctors, nurses and students) with owner consent. All cats had a complete orthopaedic examination performed by a single investigator (KLP). If any abnormality was identified, that cat was excluded from the study. All cats were weighed on the same electronic baby scale immediately before gait analysis.

Participating cats were asked to walk on the treadmill (CanidGait®, Zebris Medical GmbH, Allgäu, Germany) following an acclimatisation period of 5 to 10 minutes (Schnabl‐Feichter et al., 2020). Three valid trials were collected, with each trial consisting of 10 complete gait cycles (Söhnel et al., 2022). Positive reinforcement such as toys, treats, food and owner presence was utilised to encourage participation. If at any time a cat refused to walk following an appropriate acclimation period, it was excluded from the study. Following completion of gait trials, cats were returned to their owners.

Outcome measures were PVF and VI normalised to % body weight (N and Ns, respectively), SI (%) and time to record three valid trails (minutes). The SI was calculated as previously described (Schnabl‐Feichter et al., 2017, 2020), where an SI of 0% would represent perfect symmetry between contralateral limb pairs (left vs. right). Step length (cm) and stance phase duration (%) were also recorded. Owner consent was obtained prior to presentation for orthopaedic examination *via* a signed consent form. Verbal consent was obtained prior to gait analysis. All owners were present for gait analysis.

### Pressure‐sensitive treadmill set up

**FIG 1:**
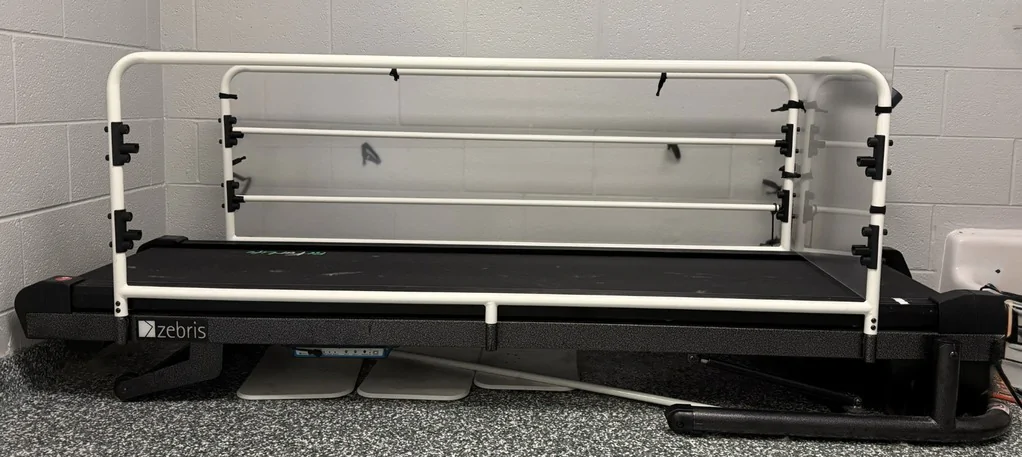
Gaiting set‐up for the pressure‐sensitive treadmill (PST). The PST had a specified computer which was connected to both the treadmill and video camera to record and synchronise data.

Measurements took place in a dedicated gait room at Michigan State University (Fig 1). No calibration was required for the PST as it is pre‐set by the company. Based on preliminary pilot data, the velocity of the PST was set to 0.44 m/s as this was the velocity most cats preferred to walk at without being “hunched,” taking normal strides and without running or “riding” the treadmill backward. Clear plexiglass safety panels were placed over the open side of the treadmill to keep cats from jumping off and a dedicated person stood at the back of the treadmill during all gaiting to ensure cat safety.

**FIG 2:**
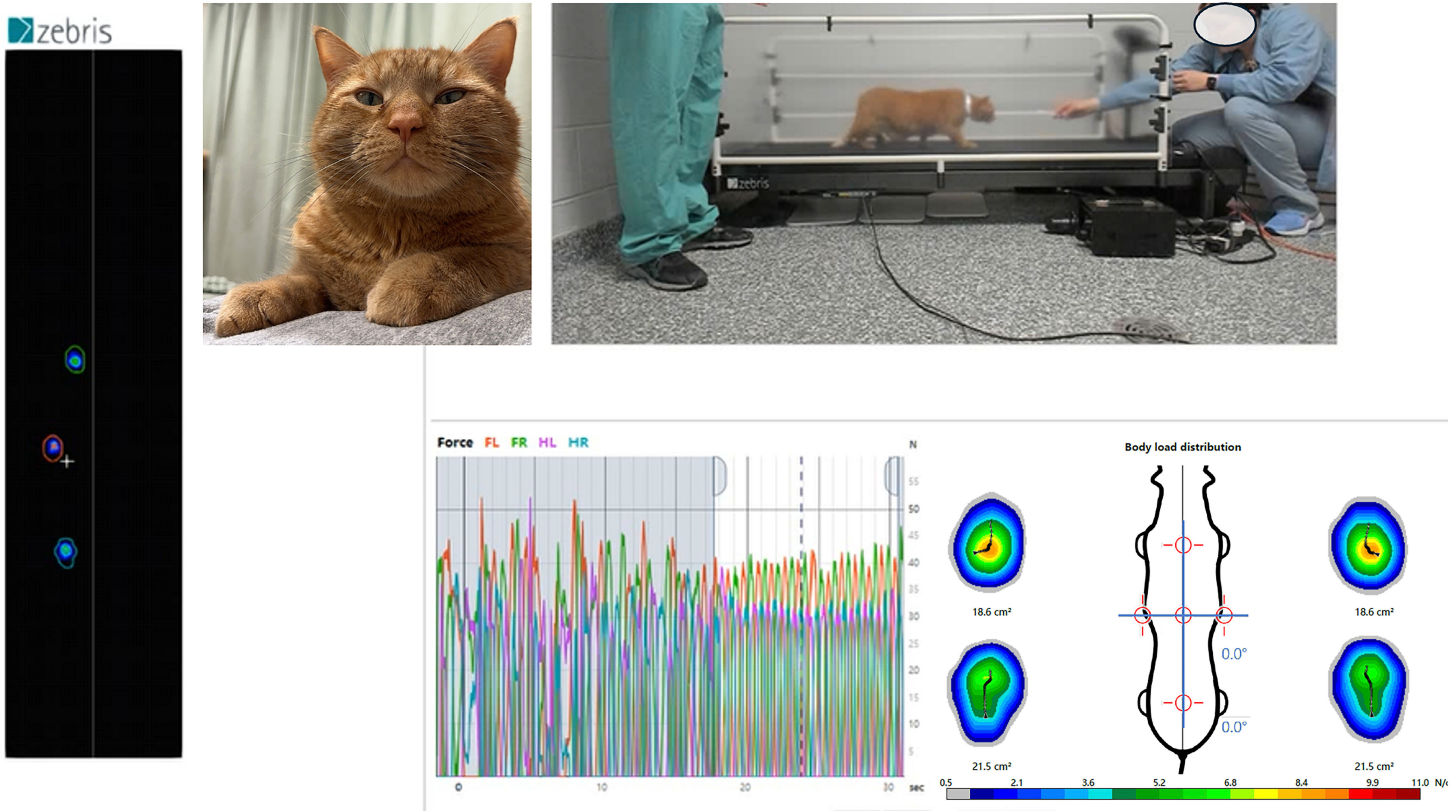
Synchronised video recording output. Foot fall data and force were captured every 30 seconds until three valid trials were completed. The synchronised video was used to verify correct foot labelling while the cat was walking. Body skew can be seen as 0° indicating a symmetrical gait in this representative image.

The cat was placed on a stationary treadmill initially. Incremental slow increases (0.03 m/s at a time) in velocity were implemented until the cat was walking at the desired 0.44 m/s at which point data collection was started with a sampling rate of 100 Hz. Synchronised video recordings correlating with foot fall data and force were captured (Fig 2) every 30 seconds until three valid trials were completed.

### Statistical analysis

The mean ± standard deviation of SI and trial time were evaluated for normality using a Kolmogrov–Smirnov test. Descriptive results including PVF (N), VI (Ns), stance phase duration (SPD – the period of the gait cycle from initial paw strike to “toe‐off”), step length (SL), ease of system use and cat willingness to walk were recorded. Following normality testing, a student's *t*‐test was used to compare left and right limb pairs in SPD and SL with significance set at *P* < .05.

## RESULTS

Sixty‐six cats were presented for study inclusion. Of these, seven cats were excluded due to abnormalities detected on orthopaedic examination. During gait analysis, a further nine cats were unwilling to walk on the PST and were excluded.

A total of 50 cats were included in the study, 31 males and 19 females. The mean age of the cats was 4.9 ± 3.6 years (range: 8 months to 14 years). The mean body weight was 4.9 ± 1.1 kg (range: 2.9 to 8.6 kg).

### Temporospatial parameters (Tables 1 and 2)

**Table 1:** Mean (SD) values for PVF and VI for all 50 cats

Variable	Limb	Treadmill (PST)
PVF (% BW, N)	Right forelimb	57.89 ± 4.45
	Left forelimb	57.48 ± 3.65
	Right hindlimb	46.44 ± 3.27
	Left hindlimb	46.28 ± 3.92
VI (% BW, Ns)	Right forelimb	27.43 ± 3.20
	Left forelimb	26.95 ± 3.65
	Right hindlimb	21.1 ± 3.27
	Left hindlimb	20.91 ± 2.91

No significant (*P* > .05) differences between data generated from right and left limb pairs were observed

PVF Peak vertical force, VI Vertical impulse, PST Pressure sensitive treadmill

**Table 2:** Temporospatial parameters for all 50 cats (male and female)

Variable	Right forelimb	Left forelimb	Right hindlimb	Left hindlimb
SPD (%)	65.23 ± 3.64		61.23 ± 2.7	
SL (cm)	21.59 ± 2.51	22.15 ± 2.35	21.64 ± 2.23	22.39 ± 2.33

SPD is reported as right and left side combined, while step length is reported as individual right and left side

SPD Stance phase duration, SL Step length

The mean velocity was 0.45 ± 0.03 m/s. No significant differences were noted between right and left limb pairs (*P* > .6 for all).

**FIG 3:**
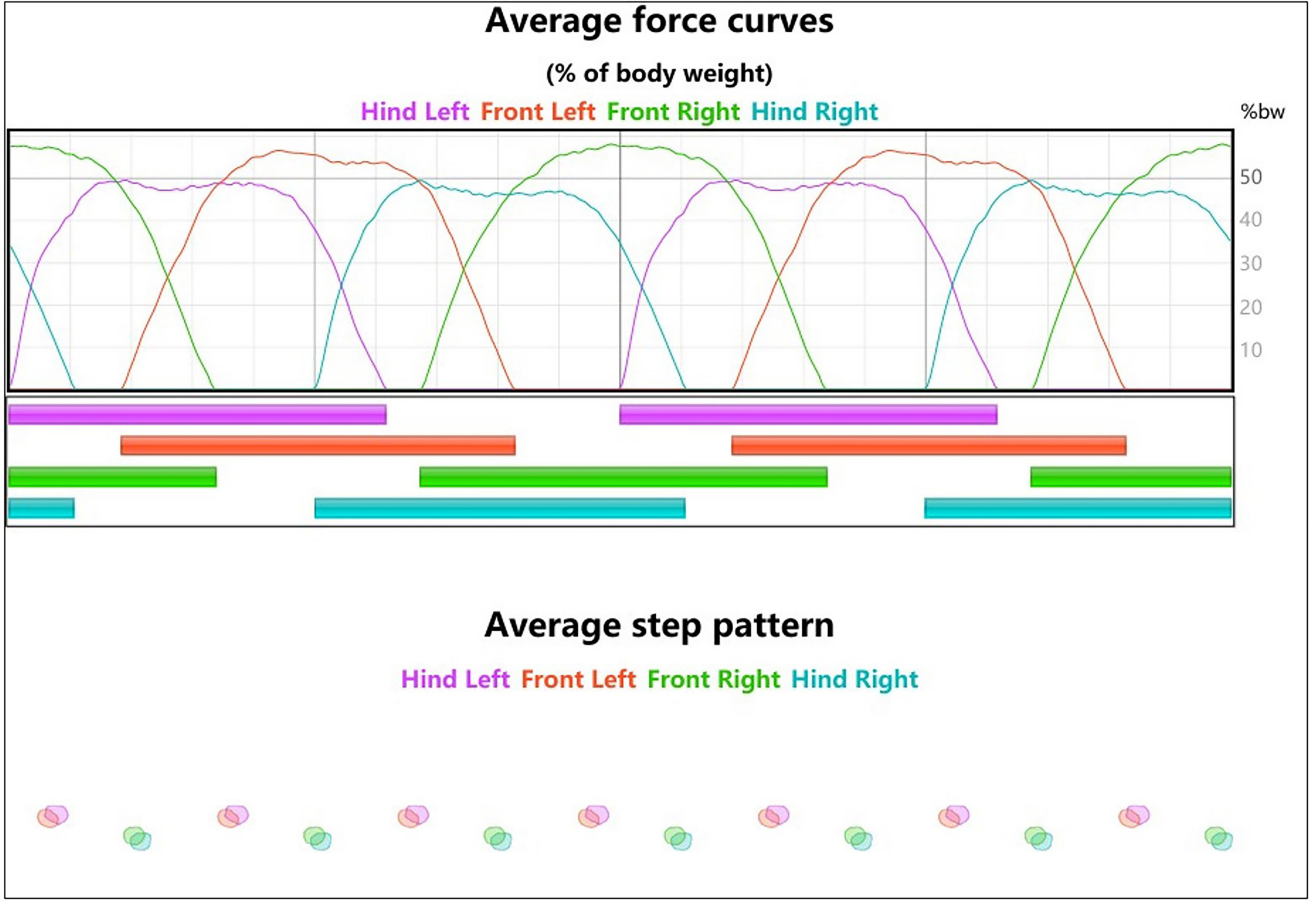
Force curves and step pattern data output from the PST software. A symmetrical gait can be seen with the typical “m” shaped pattern. The first peak represents the peak vertical forces (PVF) associated with initial paw strike and the second peak represents the PVF generated in the late stance phase associated with propulsion.

Mean PVF of the forelimbs was 57.5 ± 4.2 N (right 57.9 ± 4.5 N and left 57.5 ± 3.7 N) and for hindlimbs was 46.4 ± 3.8 N (right 46.4 ± 3.3 N and left 46.3 ± 3.9 N). Average representative force curves can be seen in Fig 3. Mean VI for the forelimbs and hindlimbs was 27.2 ± 3.4 Ns and 21.0 ± 3.1 Ns, respectively. Right versus left forelimb VI was 27.4 ± 3.2 Ns and 26.9 ± 3.7 Ns; in the hindlimbs, these values were 21.1 ± 3.3 Ns (right) and 20.9 ± 2.9 Ns (left).

For SPD and SL, there were no significant differences between left and right limb pairs. The SPD of the forelimbs was 65.2 ± 3.6% of the gait cycle. The SL for the forelimbs was 21.9 ± 2.4 cm. There was a significant difference between males and females, with male step length at 22.5 ± 2.5 cm and females at 20.8 ± 2.0 cm (*P* < .001). In the hindlimbs, the SPD was 61.2 ± 2.7%, while the SL was 22.0 ± 2.3 cm. The difference between males and females was significant (*P* < .001), but the difference between forelimbs and hindlimbs was not (*P* = .67).

### Symmetry index

**FIG 4:**
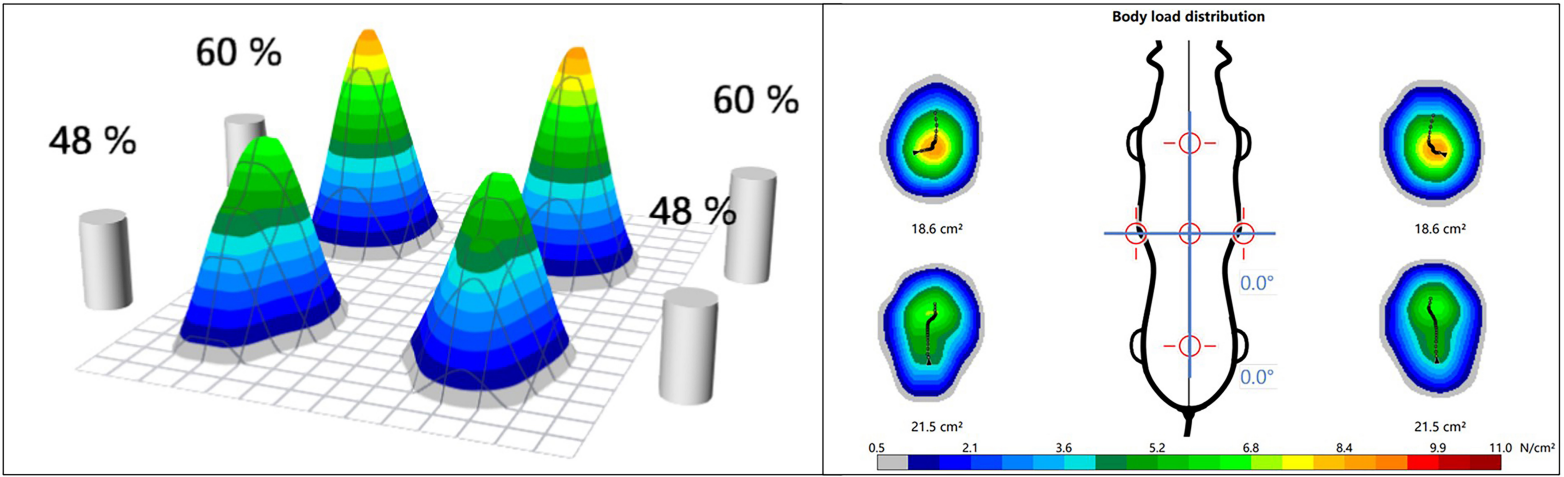
Force and body load distribution graphs showing a symmetrical gait (contralateral forelimb and hindlimb distribution is identical).

The SI of the forelimbs was 1.0 ± 0.1% and of the hindlimbs was 1.0 ± 0.1%. Fig 4 shows a representative image of the SI and body weight distribution report from one of the 50 cats included in the study.

### Data collection times

The mean data collection time was 3.5 ± 1.5 minutes. To expand further, in a single 10‐second period, 12 to 14 complete gait cycles were recorded on the PST.

## DISCUSSION

This study showed that it was possible to obtain valid kinetic data using the PST. When evaluating the PVF values, cats placed ~55% to 60% of their body weight on the forelimbs and ~40% on the hindlimbs, which is consistent with reports from prior studies (Schnabl & Bockstahler, 2015; Schnabl‐Feichter et al., 2017). An important consideration in gait analysis and obtaining accurate PVF and VI is the velocity of the subjects. This was consistent across cats using the PST, with a standard deviation of 0.03 m/s. This highlights one of the main benefits of the PST system; in that, speed can be tightly controlled across subjects (which is especially important in cats that are not walked on a leash). Previous studies evaluating GRFs in normal cats have reported velocities of between 0.3 and 0.7 m/s (Lascelles et al., 2007; Verdugo et al., 2013). One group of investigators specifically noted the difficulty in getting cats to walk in a straight line at a constant speed (Verdugo et al., 2013). The set PST speed in the current study (0.44 m/s) falls within previously reported ranges and was chosen based on pilot data to represent a walking gait where cats felt comfortable, were not panicked and moved without crouching. Therefore, the authors' feel it is an accurate representation of a normal, clinically relevant cat walking gait. Furthermore, it was readily repeatable across all 50 participants of varying temperaments. However, the effect of different travelling speeds was not investigated in this study, and future investigations evaluating cats moving at faster speeds on the PST are warranted.

When considering the standard deviations of all GRFs, there was minimal variation with the PST system. This finding is expected given the known effect of trial repetition on data variation reported by Jevens and confirmed by Söhnel (Jevens et al., 1993; Söhnel et al., 2022). This highlights a second benefit of the PST system, especially for cats, which are recognised to be impatient, easily bored and often not as accustomed to restraint or handling as dogs typically are. Clinically, the PST may be more useful in such a scenario because the data collection process is streamlined, which could be valuable, particularly with cats that have a shorter tolerance period.

The authors' also noted a third benefit to the PST, which was ease of data collection for cats weighing <4 kg. Interestingly, while a previous study reported that the PST was designed for dogs with body weights of 5 to 80 kg, the lightest cat in the present study was 2.9 kg with no difficulties being encountered during data collection. Clinically, this feature will allow a much larger population of cats to be included for a given study, as well as reducing the time for investigators to collect valid trials in these patients.

Temporospatial parameter results were as expected based upon previous studies. As with earlier reports of kinetic analysis in normal cats, our results did not show differences between left and right contralateral limb pairs. Additionally, our results agreed with reports evaluating treadmills compared to stationary equipment, with cats having a higher stance phase duration when walking on the treadmill (Assaf et al., 2019; Söhnel et al., 2022). Furthermore, step lengths were consistent using the PST, which the authors' attribute to a consistent travelling speed among cats.

Symmetry indices of the forelimb and hindlimb pairs confirmed the findings of the normal orthopaedic exams, with values around 1%. Previously, Söhnel et al. reported that the PST was excellent for identifying limb asymmetry (Söhnel et al., 2022), and our results confirm this finding. Clinically, when combined with an orthopaedic exam, use of the PST may be able to help localise lameness to a particular limb, which could be of diagnostic benefit considering typical cat behaviour in hospital (hiding, slinking or cautious/slow walking).

The current study demonstrated rapid data collection times. Given the mean of 3.5 minutes, it is infinitely possible to consider including use of the PST as part of routine outcomes assessment on the clinic floor. Regardless of setting, this would fit within a typical appointment time slot and could be performed prior to sedation for any diagnostic imaging or other treatments. Additionally, cats seemed to tolerate well the regularly moving walkway of the treadmill, and in a 10‐second time interval, approximately 12 to 14 complete gait cycles were captured. As previously mentioned, this could be a benefit for a typically challenging species to work for gait analysis clinically. This tool will also likely facilitate future studies on feline gaiting, a topic which has historically only sporadically been investigated. Indeed, as of 2015 when a meta‐analysis on feline GRFs was performed, there were only 12 available studies for review (Schnabl & Bockstahler, 2015).

Of the 66 cats presented for study inclusion, nine were excluded due to their unwillingness to walk on the PST. This is likely reflective of what clinicians could expect from their patients when using this system (a 14% exclusion rate) and should be a consideration when designing a study. Furthermore, patients were only allotted up to 10 minutes of acclimatisation time to maintain homogeneity across subjects. However, it is possible that, with additional time, cats may have been willing to participate on the PST, and this could still be accommodated in an efficient manner given the short duration needed for data collection specifically.

Another important consideration for implementing gait analysis into clinical practice is the available space. The PST measures 223 cm long by 61 cm wide, with an additional width of 183 cm for the motion capture camera. It can also be stored in an upright position when not in use if necessary. If limited space is available, clinicians may consider the PST as an alternative option to a PSW system.

Some limitations must be acknowledged. First, results may vary with use of different gaiting systems and our results should not be extrapolated to any alternative options. Similarly, results may vary with different speeds, and further studies are warranted to evaluate the effect of this on the new PST. Second, all cats were volunteered by the veterinary medicine community at our institution, potentially biasing the degree of cat cooperation since veterinary‐owned animals may be more comfortable in hospital settings, and owners likely would not volunteer a non‐cooperative cat. Additionally, all cats were intentionally selected to have no orthopaedic abnormalities; results may vary for clinically lame patients. Finally, orthopaedic exams were performed by a single experienced examiner, and results corroborated the exam findings in this population. Varying results may be obtained with less experienced examiners or less cooperative cats.

In conclusion, the results from the current study support the use of the PST for kinetic gait evaluation in cats. Indeed, the PST produced consistent and repeatable data, was easy and fast to use, and cats readily participated in most cases. The homogeneity of protocols using this system could allow for useful comparisons between studies, hospitals and patients in future investigations. Furthermore, it may be a feasible option for clinicians interested in gait analysis with limited space available for set‐up.

### Author contributions

**D. M. Marturello:** Study conception, study design, data collection, data reduction, abstract presentation, primary manuscript writing and revision. **K. B. de Sousa:** Data collection, manuscript revision approval. **B. L. Boger:** Data collection, manuscript revision approval. **S. A. Shull:** Facilities and equipment supply, data collection secondary, manuscript revision approval. **K. L. Perry:** Study design, data collection, manuscript writing (secondary), manuscript revision approval.

### Conflict of interest

No conflicts of interest have been declared. Dr Perry is an associate editor of Journal of Small Animal Practice and co‐author of this article. They were excluded from editorial decision‐making related to the acceptence of this article for publication in the journal.

## Data Availability

The data that support the findings of this study are available on request from the corresponding author. The data are not publicly available due to privacy or ethical restrictions.
